# Deficient Event-Related Theta Oscillations in Individuals at Risk for Alcoholism: A Study of Reward Processing and Impulsivity Features

**DOI:** 10.1371/journal.pone.0142659

**Published:** 2015-11-18

**Authors:** Chella Kamarajan, Ashwini K. Pandey, David B. Chorlian, Niklas Manz, Arthur T. Stimus, Andrey P. Anokhin, Lance O. Bauer, Samuel Kuperman, John Kramer, Kathleen K. Bucholz, Marc A. Schuckit, Victor M. Hesselbrock, Bernice Porjesz

**Affiliations:** 1 SUNY Downstate Medical Center, Brooklyn, NY, United States of America; 2 Washington University School of Medicine, St. Louis, MO, United States of America; 3 University of Connecticut Health Center, Farmington, CT, United States of America; 4 University of Iowa, Iowa City, IA, United States of America; 5 University of California San Diego, San Diego, CA, United States of America; Mayo Clinic, UNITED STATES

## Abstract

**Background:**

Individuals at high risk to develop alcoholism often manifest neurocognitive deficits as well as increased impulsivity. Event-related oscillations (EROs) have been used to effectively measure brain (dys)function during cognitive tasks in individuals with alcoholism and related disorders and in those at risk to develop these disorders. The current study examines ERO theta power during reward processing as well as impulsivity in adolescent and young adult subjects at high risk for alcoholism.

**Methods:**

EROs were recorded during a monetary gambling task (MGT) in 12–25 years old participants (*N* = 1821; males = 48%) from high risk alcoholic families (HR, *N* = 1534) and comparison low risk community families (LR, *N* = 287) from the Collaborative Study on the Genetics of Alcoholism (COGA). Impulsivity scores and prevalence of externalizing diagnoses were also compared between LR and HR groups.

**Results:**

HR offspring showed lower theta power and decreased current source density (CSD) activity than LR offspring during loss and gain conditions. Younger males had higher theta power than younger females in both groups, while the older HR females showed more theta power than older HR males. Younger subjects showed higher theta power than older subjects in each comparison. Differences in topography (i.e., frontalization) between groups were also observed. Further, HR subjects across gender had higher impulsivity scores and increased prevalence of externalizing disorders compared to LR subjects.

**Conclusions:**

As theta power during reward processing is found to be lower not only in alcoholics, but also in HR subjects, it is proposed that reduced reward-related theta power, in addition to impulsivity and externalizing features, may be related in a predisposition to develop alcoholism and related disorders.

## Introduction

Electrical activity of the human brain, termed electroencephalogram (EEG), was first recorded by the German physiologist and psychiatrist Hans Berger (1873–1941) in 1924 (cf. [1]). The EEG activity recorded during specific cognitive events has been studied either in the time domain as event-related potentials (ERPs) comprising trial-averaged waveforms, or in the frequency domain as event-related oscillations (EROs) with time-frequency characteristics. According to Basar [2], specific frequencies of EROs underlie different cognitive functions, and selectively distributed delta, theta, alpha and gamma oscillatory systems act as resonant communication networks through large populations of neurons during cognitive processing. ERO signals with phase-alignment across the trials of cognitive events are termed ‘evoked’ or ‘phase-locked’ oscillations, while the signals that are ‘out-of-phase’ are ‘induced’ or ‘non-phase-locked’ oscillations [3]. The ‘total’ ERO power consists of both evoked and induced signals [4].

ERO methods have been effectively implemented to investigate cognitive processing in healthy individuals [5–14] as well as in a variety of clinical conditions [15], including alcohol use disorders (AUDs) [16]. AUD is a common disorder with complex etiology involving genetic and environmental influences and their interactions (cf. [17]). Several neurocognitive dysfunctions, reflecting impairments in several brain regions and/or neural circuitries, have been associated with AUDs. In recent years, several studies have employed EROs to investigate brain dysfunction in individuals with (chronic) alcoholism as well as in those with a predisposition to develop alcoholism (for a review, see Pandey et al. [16]).

Over the past few decades, studies have reported electrophysiological abnormalities related to resting state and during cognitive processing in individuals with AUDs and in high risk (HR) subjects with a family history of alcoholism (for reviews, see [17–19]). Dysregulation in reward systems is a prominent dysfunction in alcoholism [20–22], and electrophysiological studies during gambling paradigms have revealed reward processing dysfunction in alcoholics and HR offspring [23–26]. ERP studies of reward processing have frequently employed monetary gambling tasks and examined two major components: a negative going component around 200–250 ms called the outcome- or feedback-related negativity (ORN or FRN or N2), and a positive going component at about 300–500 ms called the outcome- or feedback-related positivity (ORP or FRP or P3) [27–42]. These outcome-related ERP components have been shown to be predominantly composed of theta oscillations [14,30,39,43–45] that mediate aspects of cognitive processing [14,15,46–60], and have been implicated in several neuropsychiatric disorders (for reviews, see [15,61–63]), including alcoholism [4,16,49,64–66].

While the electrophysiological signatures of reward processing have been well-established, the reward processing mechanisms that may mediate alcohol dependence and its risk have not yet been fully understood, as there are only a few ERP/ERO studies that have examined reward processing in alcoholics and their high risk offspring. In 2008, using the Balloon Analogue Risk Task (BART), Fein and Chang [24] reported smaller FRN in treatment-naive alcoholics from families densely affected with alcohol problems. Using a monetary gambling task, our group reported that alcoholic subjects showed smaller outcome related ERP components (ORN/N2 and ORN/P3) [25] as well as decreased theta power during reward processing [49] compared to controls. During reward processing, alcoholics were also found to manifest increased impulsivity and negative correlations between impulsiveness and P3 amplitude [25] as well as between impulsiveness and theta power [49]. Recently, we conducted an ERP study in adolescents and young adult offspring from alcoholic families (HR, high risk to develop alcoholism who had at least one parent with alcohol dependence) and found that adolescent females and young adult males showed reduced P3 amplitudes during the loss conditions [67] but no difference in the gain conditions compared to low risk offspring from control families (LR) who had no parental history of alcoholism. It is surprising that very few studies have examined the reward related ERP components (ORN and ORP) as well as the theta EROs underlying these components in the realm of alcoholism [24,25,49,67]. Further, reward related theta oscillations, a primarily constituent of these ERP components, have not yet been studied in high risk offspring of alcoholics, while this measure has been explored in normal subjects [14,30,39,43,44] and alcoholics [49] during reward processing.

The current study is the first to examine ERO theta power during reward processing in a high risk sample, and has used a large sample of adolescent and young adult HR offspring from high density alcoholism families in the Collaborative Study on the Genetics of Alcoholism (COGA). The present study is an extension of our previous work, and seeks to determine: (i) whether lower reward related ERO theta power and increased impulsivity observed in alcoholics [49] is also present in HR compared to LR subjects; and (ii) whether HR subjects, who manifested lower P3 amplitude during reward processing in our previous study [67], would also show decreased reward related ERO theta power underlying the P3 [68,69] wave while evaluating loss and gain. Specifically, the current study aims to address whether (i) deficits in the same measure (lower ERO theta power) that has been observed in alcoholics is also observed in HR subjects, and (ii) the same group (HR subjects) shows deficits in both measures (P3 and EROs) during reward processing. Although theta oscillatory activity during reward processing has been studied in healthy individuals [39,44,70] and in alcoholics [49], the present study is the first to examine event-related theta activity during reward processing in a monetary gambling task in subjects who are at high risk for alcoholism. Using the same approach as in our previous studies of reward processing [49,67], the present study also examined current source density (CSD), to analyze the cortical sources of the surface potentials [71] during theta EROs. The study has compared psychometrically assessed impulsivity as well as externizing diagnoses, such as substance use disorders, conduct disorder (CD), antisocial personality disorder (ASPD), attention deficit hyperactivity disorder (ADHD), and oppositional defiant disorder (ODD), between the risk groups (i.e., HR and LR). We expected that HR compared to LR offspring would manifest lower ERO total theta power and decreased CSD activations in both loss and gain conditions, along with increased impulsivity scores and higher prevalence rates of externalizing disorders. Therefore, findings from the current study may further characterize ERO theta power during reward processsing in individuals at risk and in addition to externalizing characteristics, including impulisivity, may serve as a potential marker for a predisposition to develop alcoholism and related disorders.

## Methods

### Ethics Statement

The experimental protocols were approved by Institutional Review Board (IRB) of each participating centers of COGA: University of Connecticut; Indiana University; University of Iowa; SUNY Downstate; Washington University in St. Louis; University of California at San Diego; Rutgers University; University of Texas Health Science Center at San Antonio, Virginia Commonwealth University, Icahn School of Medicine at Mount Sinai, and Howard University. Data were collected from the first six of these eleven centers. All participants signed an IRB approved written consent form. Parents or legal guardians signed for their minor children. Consent was obtained by research personnel who had adequate training and competency in ethical guidelines and consent process. Study procedure, risks and benefits were explained to each participant and/or the parent/guardian before collecting data. It was also ascertained that participants were not disadvantaged in any way by not participating in the study.

### Participants

The sample included 1821 adolescents and young adults (874 males and 947 females) between 12 to 25 years of age (see Table 1) and was derived from the prospective sample [72] of the COGA study [73,74]. The sample included offspring of the high-risk (multiple alcohol dependent adult family members) and comparison (community) families ascertained in previous phases of COGA. Participants are reassessed every two years with clinical, behavioral and neurophysiological assessments. Data from the six collection centers have been included in this study. Recruitment and assessment procedures have been described elsewhere [73,75–77], and are also available at this website: https://zork5.wustl.edu/coganew/data/instruments.html. Details regarding access to COGA data are available through the National Institute of Alcoholism and Abuse at http://www.niaaa.nih.gov/research/major-initiatives/collaborative-studies-genetics-alcoholism-coga-study#Access. COGA data are also available from the publically accessible dbGAP database at http://www.ncbi.nlm.nih.gov/gap/?term=COGA [IDs: phs000092.v1.p1, phs000125.v1.p1, and phs000763.v1.p1].

**Table 1:** Number of participants and age (mean and SD) categorized by age group and gender across risk groups (HR and LR). Mean ages between LR and HR groups have been compared using t-tests.

Gender	Age Group	LR			HR			*t*-value	*p*
		*N*	Age		*N*	Age			
			Mean	SD		Mean	SD		
**Male**	**12–15**	67	13.34	1.28	319	13.64	1.20	-1.775	0.0737
**Male**	**16–25**	62	18.85	1.95	426	19.28	2.20	-1.475	0.1408
**Female**	**12–15**	97	13.48	1.18	334	13.67	1.24	-1.329	0.1845
**Female**	**16–25**	61	19.22	2.19	455	19.51	2.16	-0.979	0.3281

Subjects were instructed to refrain from using alcohol and substances for 5 days prior to EEG recording. Subjects were excluded from neurophysiological assessment if they had any of the following: (1) report of recent drug/alcohol use or a positive breath-analyzer test, (2) hepatic encephalopathy/cirrhosis of the liver, (3) history of head injury, seizures or neurosurgery, (4) uncorrected sensory deficits, (5) history/symptoms of psychoses, (6) self-reported positive test for human immunodeficiency virus, and/or (7) other acute/chronic medical illnesses that affect brain function. The HR group consisted of individuals from the COGA high density alcoholism families who had at least one parent with DSM-IV alcohol dependence, while the participants for the LR group consisted of individuals from the community families without any parental history of alcohol dependence. The current study is cross-sectional by design and involves data from the initial (first) assessment only. The groups were further subdivided based on gender and age group (12–15 and 16–25 years old) (see Table 1). These age ranges were adapted in order to maintain proportionally optimal sample size in each subgroup. Although unequal interval of age range in these groups was due to skewness in the sample, with more subjects represented at younger ages, these age ranges provided statistical power to investigate differences in younger and older groups. The HR and LR groups did not differ in sample distribution in terms of mean age (see Table 1) and gender (χ^2^ = 1.27; *p* = 0.2741). Table 2 shows prevalence rates of lifetime diagnoses for externalizing disorders for HR and LR groups in males and females. The prevalence rates were significantly higher in HR subjects compared to LR individuals in several diagnoses for each gender and in the combined sample.

**Table 2:** Prevalence rates in counts and percentage (in parentheses) for the diagnoses of externalizing disorders (EXT) in LR and HR groups for each gender and for total sample. Significance levels based on Chi-square tests (Pearson χ^2^ or Likelihood Ratio) have been marked with asterisks (in HR columns). Empty cell (with a dash) means the count of zero.

Diagnoses (DSM-IV)	Male		Female		Total	
	LR	HR	LR	HR	LR	HR
Alcohol Dependence	2 (1.55)	20 (2.69)	1 (0.63)	28 (3.55)*	3 (1.05)	48 (3.13)*
Alcohol Abuse	7 (5.43)	92 (12.37)*	4 (2.53)	62 (7.86)**	11 (3.83)	154 (10.04)***
Tobacco Dependence	5 (3.88)	64 (8.60)*	2 (1.27)	62 (7.86)***	7 (2.44)	126 (8.21)***
Marijuana Dependence	6 (4.65)	100 (13.44)**	3 (1.90)	59 (7.48)**	9 (3.14)	159 (10.37)***
Marijuana Abuse	4 (3.10)	56 (7.53)*	1 (0.63)	33 (4.18)**	5 (1.74)	89 (5.80)***
Cocaine Dependence	-	7 (0.94)	-	7 (0.89)	-	14 (0.91)*
Cocaine Abuse	-	2 (0.27)	-	1 (0.13)	-	3 (0.20)
Stimulant Dependence	-	5 (0.67)	-	11 (1.39)*	-	16 (1.04)*
Stimulant Abuse	-	1 (0.13)	-	2 (0.25)	-	3 (0.20)
Sedative Dependence	-	1 (0.13)	-	3 (0.38)	-	4 (0.26)
Sedative Abuse	-	2 (0.27)	-	1 (0.13)	-	3 (0.20)
Opiate Dependence	-	1 (0.13)	-	5 (0.63)	-	6 (0.39)
Opiate Abuse	-	4 (0.54)	-	4 (0.51)	-	8 (0.52)
Other Drug Dependence	-	6 (0.81)	-	8 (1.01)	-	14 (0.91)*
Other Drug Abuse	-	2 (0.27)	-	4 (0.51)	-	6 (0.39)
ASPD	3 (2.33)	39 (5.24)	1 (0.63)	25 (3.17)	4 (1.39)	64 (4.17)
CD	5 (3.88)	80 (10.75)**	5 (3.16)	45 (5.70)	10 (3.48)	125 (8.15)**
ADHD	3 (2.33)	27 (3.63)	2 (1.27)	13 (1.65)	5 (1.74)	40 (2.61)
ODD	1 (0.78)	21 (2.82)	1 (0.63)	13 (1.65)	2 (0.70)	34 (2.22)*
Any EXT disorder	21 (16.28)	262 (35.22)***	16 (10.13)	203 (25.73)***	37 (12.89)	465 (30.33)***

*p < 0.05

**p < 0.01

***p < 0.001

### Assessment Tools

The clinical and impulsivity data of the sample were collected with three COGA instruments: (1) Semi Structured Assessment for the Genetics of Alcoholism (SSAGA) [78] evaluated clinical diagnoses and symptoms; (2) Family History Assessment Module (FHAM) [79] assessed Axis I psychiatric disorders, including AUDs and related disorders, among relatives of the participants as assessed from other relatives; and (3) Barratt Impulsiveness Scale (BIS) [80] provided ratings of impulsivity in three categories viz., attentional, motor, and non-planning, as well as total scores. Cognitive/attentional impulsivity involves making quick/hasty decisions, motor impulsivity represents acting without thinking, and non-planning impulsivity indicates a lack of “futuring” or forethought [81].

### Monetary Gambling Task

**Fig 1:**
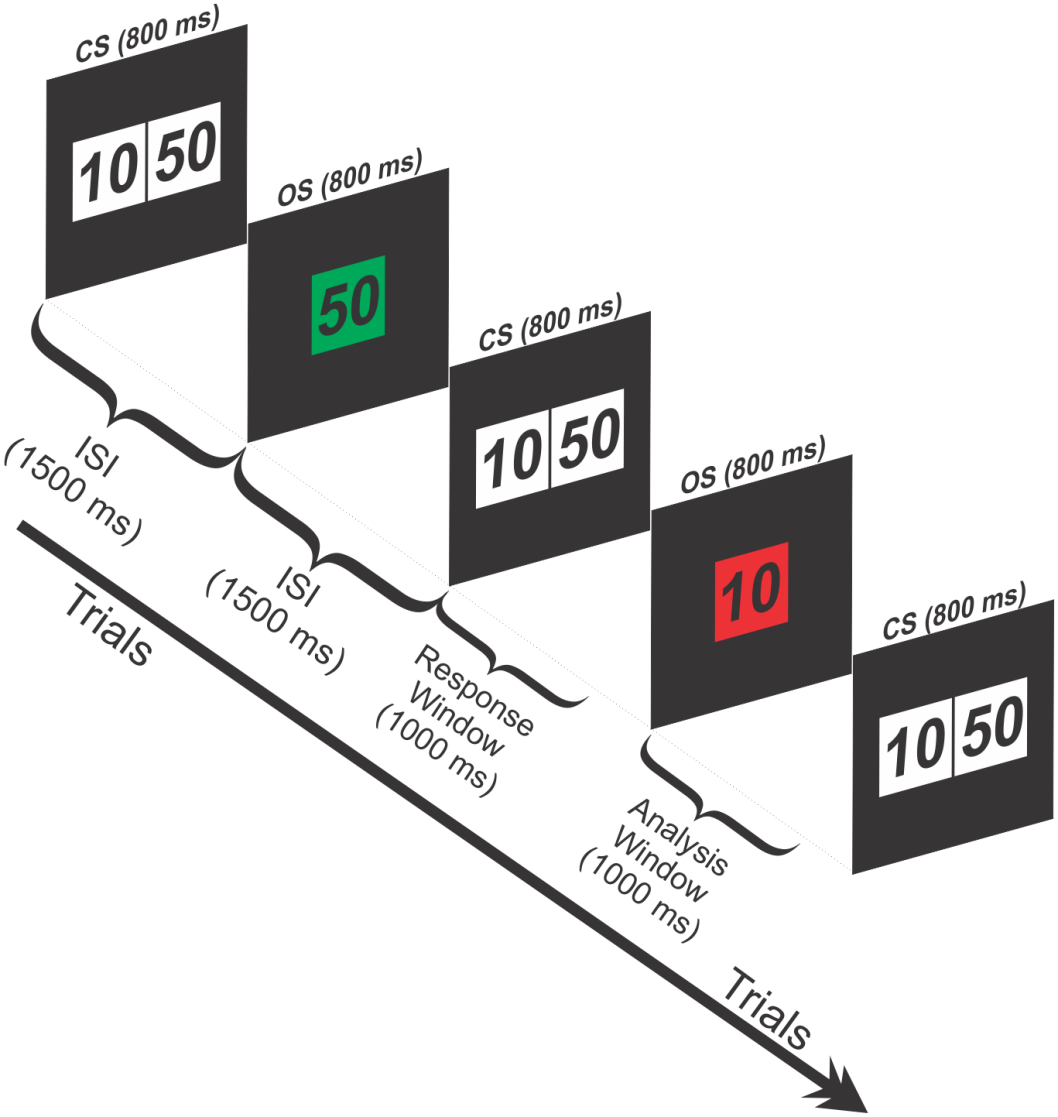
Schematic illustration of the Monetary Gambling Task (MGT). Each trial starts with a choice stimulus (CS) (white boxes) which lasts for 800 ms and displays two amounts (10¢ or 50¢). The participant selects one of the amounts with a corresponding button press. An outcome of either gain (green box) or loss (red box) for the selected amount is shown by the outcome stimulus (OS). A trial with a gain of 50¢ and the next trial with a loss of 10¢ are illustrated. The ISI between the CS and the OS is 1500 ms. Participants were required to respond to the OS within 1000 ms (i.e., response window) by selecting one of the two amounts. ERO analysis was performed on trial epochs of 1000 ms post-stimulus period after the onset of the OS (i.e., analysis window).

The monetary gambling task (MGT) used in this study is illustrated in Fig 1. Each trial began with a choice stimulus (CS), with two numbers 10¢ (left box) and 50¢ (right box) displayed for 800 ms. The participants selected a bet of either 50¢ or 10¢, and received feedback of either loss or gain for the selected amount (outcome stimulus, OS). The task details have been described in our previous publications [14,25,41,49]. The inter-stimulus interval (ISI) between a CS and OS, and between an OS and next CS is 1500 ms. The task involved a total of 172 trials, each with one of four possible outcomes: Loss 50¢, Loss 10¢, Gain 50¢, and Gain 10¢. Irrespective of the overall amounts chosen during the task, the probability of loss and gain outcomes were 50% (i.e., the number of ‘red’ and ‘green’ outcomes were equal), although the participants were not aware of this information. However, since the subject has the option to freely select the amount during each trial, the relative probability for each of the four outcomes (+50, −50, +10, −10) ranged from 0 to 50%. For example, if a subject selects ‘50’ in all the trials, the probabilities will be 50%, 50%, 0%, and 0% for the outcomes of +50, −50, +10, −10, respectively. Trial epochs containing the outcome stimuli (1000 ms poststimulus) representing the feedback of either loss or gain for each trial (i.e., the epochs following colored frames in Fig 1) were used for ERO signal processing. A prestimulus interval of 200 ms prior to the OS was used for the ERP/ERO preprocessing and/or analyses. The trials were presented in two equal size blocks, and the subjects were given a feedback of the cumulative amount lost or gained at the end of each block. At the end of the experiment, participants were paid a constant amount (irrespective of their net loss or gain) for their participation.

### EEG Data Acquisition and Preprocessing

**Fig 2:**
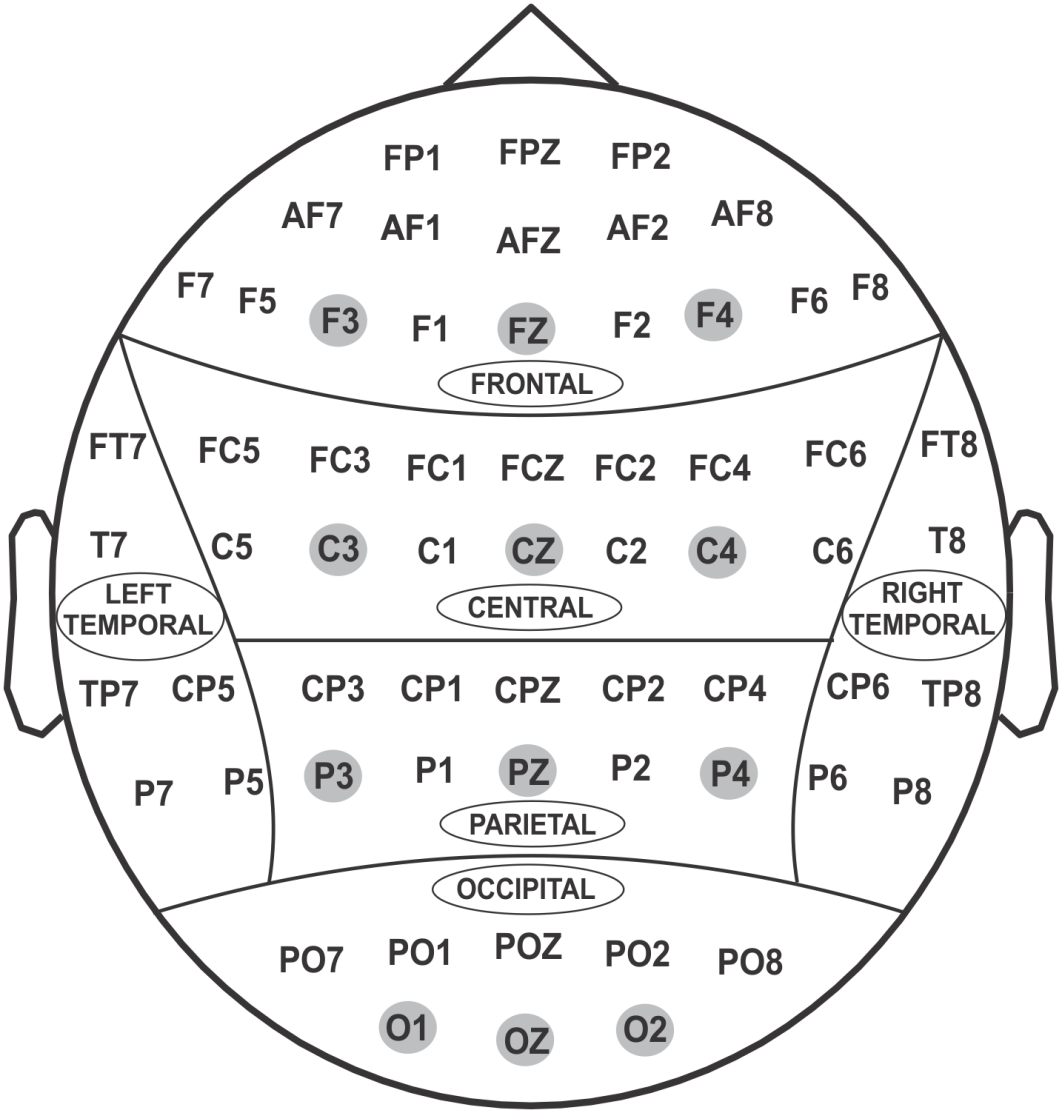
Sixty-one electrodes using extended 10–20 system as recorded in the current study. Twelve electrodes (F3, FZ, F4, C3, CZ, C4, P3, PZ, P4, O1, OZ, O2) representing frontal, central, parietal and occipital regions were selected for statistical analyses (shaded).

Identical experimental procedures and EEG acquisition systems were used at all neurophysiology collection sites of COGA [73,74] with high inter-laboratory consistency in recordings [82–85]. Subjects were seated comfortably 1 m from a monitor in a dimly lit sound-attenuated RF-shielded booth (Industrial Acoustics, Inc., Bronx, NY, USA), and wore a 61-channel electrode cap (Electro-Cap International, Inc., Eaton, OH, USA) based on the Extended 10–20 Systems [86–88] (Fig 2), with a reference electrode at the tip of the nose and with a ground electrode at the forehead. The electrooculogram (EOG) was recorded by a supraorbital vertical electrode and by a horizontal electrode on the external canthus of the left eye. Electrode impedances were maintained below 5 kΩ. Electrical activity was amplified 10,000 times using SynAmps2 amplifiers (Compumedics USA, Charlotte, NC) and was recorded continuously over a bandwidth of DC–100.0 Hz on a Neuroscan system (Versions 4.3–4.5; Compumedics USA, Charlotte, NC) at the sampling rate of 500 Hz. The entire EEG data was resampled offline to 256 Hz for the analyses. In this study, only ‘loss 50’ (representing the loss condition) and ‘gain 50’ (representing the gain condition) were analyzed, as majority of the subjects had more trials for the 50¢ than the 10¢ condition. The preprocessed trial epochs with waveforms exceeding ±100 μV (about a third of total trials) were excluded from the analyses.

### ERO Signal Processing using S-Transform

Time-frequency (TF) data were derived using the S-transform signal processing method, introduced by Stockwell et al. [89]. S-transform has been explained in our previous papers [14,49]. S-transform is deduced from short-time Fourier transform and continuous Wavelet transform, and has a better flexibility and utility in the processing of non-stationary and complex signals [90]. This method has been applied in several recent studies to analyze time-frequency signals of event-related oscillations [4,14,65,66,91–93]. The S-transform is considered to be a generalization of the Gabor transform [94] and an extension to the continuous wavelet transform. The S-transform generates a time-frequency representation (TFR) of a signal by integrating the signal at each time point with a series of windowed harmonics of various frequencies as follows:

$$ST(t,f)=\int_{-\infty }^{\infty }h(\tau )\frac{\left|f\right|}{\sqrt{2\pi }}e^{-\frac{{\left(t-\tau \right)}^{2}f^{2}}{2}}e^{-i2\pi f\tau }d\tau$$

where *h*(*t*)is the signal,*f* is frequency, *τ* is a translation parameter, the first exponential is the window function, and the second exponential is the harmonic function. The S-transform TFR is computed by shifting the window function down the signal in time by *τ* across a range of frequencies. The window function is Gaussian with 1/*f* ^2^ variance and scales in width according to the examined frequency. This inverse dependence of the width of the Gaussian window with frequency provides the frequency-dependent resolution. The amplitude envelope of the complex-valued S-transform TFR is calculated by taking the absolute value |*ST*(*f*,*τ*)|.

In the current study, total ERO theta power (which is a combination of both phase-locked and non-phase-locked activity) was computed from the outcome trials of loss and gain conditions. Since theta activity has been shown to underlie both N2 and P3 components of the ERP [14,69,95], theta power (3.5–7.5 Hz) within the TFR corresponding to 200–500 ms of post-stimulus time window (corresponding to N2 and P3 components of outcome-related ERPs) was processed (see, Kamarajan et al. [14] for detail). The filter setting to extract theta band included a 5^th^ order Chebyshev type I filter (two-step cascade type) with ripple factor (ε) of 0.108 and ripple attenuation (Rp) of 0.05. Only the subjects who had at least fifteen artifact-free trials for each condition were included for further analyses.

### CSD Mapping

Estimation of CSD was performed using surface Laplacians [96]. These methods are more sensitive than surface potentials to local brain sources [97] as they improve the spatial resolution of EEG [98]. The CSD methods have been widely employed in studying several neuropsychiatric disorders to understand the cortical sources underlying scalp potentials during resting state and during cognitive processing (for a review, see Kamarajan et al. [99]). The CSD maps were constructed from the Laplacian transformed data as described by Wang and Begleiter [100]. This method has also been applied in our earlier studies [41,101]. Since the recorded potential at each electrode represents the resultant contributions from several adjacent and distal sources, local sources cannot be clearly estimated [102]. The CSD transform acts as a spatial filter and provides an estimate of the local radial current density [96,102,103] and represents components of the primary neural activity in the scalp region [104]. In the present study, surface Laplacian was computed on the grand mean waveforms representing ERO total theta amplitude for each subgroup and for each task condition separately. The surface Laplacian was calculated at a scalp location based on the weighted average of the measured potentials at neighboring electrodes, where the electrodes closer to the index location get more weight. Topographic maps representing mean CSD potential within 300–500 ms (μV/r^2^, where *r* = head radius in cm) were created. Z-scores were calculated for each scalp map by keeping the mean SL/CSD values of all 61 scalp electrodes as zero (‘0’) and the values above and below the mean were represented in SD units (z-scores). Then the z-score converted CSD maps were also plotted in order to compare topographic features regardless of the amplitude differences across the maps. Positivity or the source (red/orange) in the CSD maps represents increased ERO amplitude or synchronization, whereas negativity or the sink (blue/cyan) indicates decreased amplitude or desynchronization [105].

### Statistical Analyses

Statistical analyses were performed using SPSS 21.0 (IBM Corporation, Armonk, NY). Log-transformed (natural log) ERO theta power (3.5–7.5 Hz) values derived from 12 electrodes representing 4 regions, viz., frontal (F3, FZ, F4), central (C3, CZ, C4), parietal (P3, PZ, P4), and occipital (O1, OZ, O2), were used in the analysis. A repeated measures analysis of variance (RM-ANOVA) of the general linear model (GLM) was used, involving Risk Group (HR and LR), Age Group (12–15 and 16–25 years), and Gender (male and female) as between-subjects factors, and Task condition (Loss and Gain) and Region (frontal, central, parietal, and occipital) as within-subjects factors. Although affected status (i.e., individuals with any externalizing diagnosis as shown in Table 2) was initially used as a covariate in the RM-ANOVA model, it was removed from the later analysis which is presented here, as the covariate showed neither a main effect nor any interaction effect with any factors in the model. The RM-ANOVA results were extracted from the multivariate test statistics (http://www-01.ibm.com/support/knowledgecenter/SSLVMB_20.0.0/com.ibm.spss.statistics.help/alg_glmrm_within-subjects.htm) as the ERO data for the within-subjects factors did not adhere to sphericity assumptions (i.e., the equality of the variances of the differences between levels of the repeated measures factor such as region). In other words, an appropriate alternative for the sphericity assumption while analyzing the EEG data is to use multivariate tests on the within-subjects effects within the GLM repeated measures model [106–108], as used in the current study. Further, Bonferroni adjusted pairwise comparisons within each significant main and interaction effects were analyzed using estimated marginal means (EMM) and standard errors (SE). The BIS scores between LR and HR groups were analyzed using one-way ANOVA. Chi-square tests (Pearson χ^2^) were used to determine the significance levels for the prevalence rates of externalizing diagnoses between HR and LR groups. Likelihood ratio was used when any cell in the comparison had less than 5 observations. Pearson correlations were used to analyze the relationship between BIS scores and theta power.

## Results

### Theta Power

**Fig 3:**
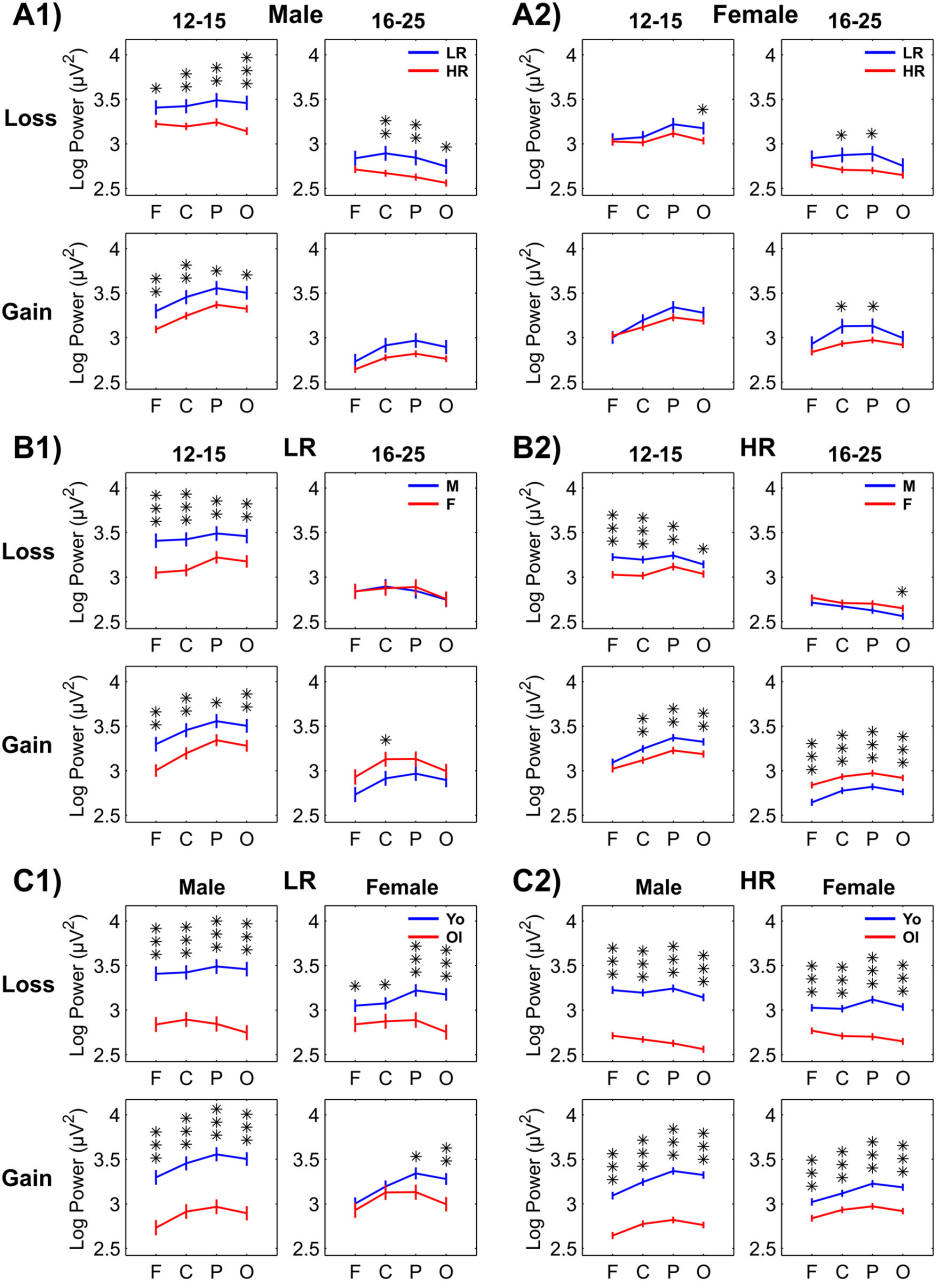
Multiple comparisons of log-transformed theta power across risk group, gender and age group. Estimated marginal means and standard error (±1 SE) of pairwise comparisons between the risk groups (panel-sets A1 and A2), gender (panel-sets B1 and B2), and age groups (panel-sets C1 and C2) in loss and gain conditions have been plotted for each scalp region (F, frontal; C, central; P, parietal; and O, occipital). Bonferroni adjusted level of significance for each comparison is shown in asterisks (**p < 0.01, ***p < 0.001). [M = Male; F = Female; Yo = Younger Age Group (12–15 years); Ol = Older Age Group (16–25 years)].

Main and interaction effects of theta power are listed in Table 3 (for all the effects of risk group as well as for the significant effects of gender and age group). The mean and SD values of log-transformed theta power for all the factors and their levels have been provided in S1 Table. Pairwise comparisons of the estimated marginal means between the levels of risk groups, gender, and age groups at each scalp region for the loss and gain conditions are illustrated in Fig 3. The results of the RM-ANOVA for the between-subjects factors (i.e., risk group, gender, and age group) as well as for the within-subjects factors (i.e., condition, and scalp region) have been summarized below. While the EMM and SE (in parentheses) have been provided only for the significant effects, mean and SD for the total sample as well as for the subgroups have been provided in the S1 Table.

**Table 3:** Main and interaction effects of theta power as elicited by the GLM RM-ANOVA model. F-values and p-values (*p < 0.05, ***p < 0.001) are shown for all the effects of risk group as well as for the significant effects of gender and age group.

Effect	*F*	*p*
Risk Group	**20.83**	<0.0001***
Risk Group × Gender	1.79	0.1805
Risk Group × Age Group	0.00	0.9652
Risk Group × Gender × Age Group	0.88	0.3493
Risk Group × Condition	1.85	0.1737
Risk Group × Gender × Condition	0.98	0.3214
Risk Group × Age Group × Condition	0.00	0.9856
Risk Group × Gender × Age Group × Condition	0.07	0.7843
Risk Group × Region	**3.75**	0.0107*
Risk Group × Gender × Region	0.93	0.4237
Risk Group × Age Group × Region	2.27	0.0786
Risk Group × Gender × Age Group × Region	0.30	0.8263
Risk Group × Condition × Region	1.43	0.2322
Risk Group × Gender × Condition × Region	0.90	0.4426
Risk Group × Age Group × Condition × Region	1.60	0.1882
Risk Group × Gender × Age Group × Condition × Region	0.68	0.5618
Gender × Age Group	**23.65**	<0.0001***
Gender × Condition	**14.93**	0.0001***
Gender × Condition × Region	**3.08**	0.0264*
Age Group	**163.81**	<0.0001***
Age Group × Condition	**13.34**	0.0003***
Age Group × Region	**12.21**	<0.0001***
Condition	**73.31**	<0.0001***
Region	**61.07**	<0.0001***
Condition × Region	**59.57**	<0.0001***

#### Risk Group

As shown in Table 3, the main effect of risk group was highly significant [*F* = 20.83; *p* < 0.0001], showing that HR subjects [2.96 (0.01)] manifested significantly lower theta power than their LR counterparts [3.10 (0.03)]. Significant risk group × region interaction effect revealed that the difference between HR and LR subjects were significant in all four regions, and that the highest difference was observed in the parietal region [HR = 3.01 (0.01); LR = 3.18 (0.03); *F* = 22.92; *p* < 0.0001], followed by central [HR = 2.96 (0.01); LR = 3.12 (0.03); *F* = 21.92; *p* < 0.0001], occipital [HR = 2.95 (0.01); LR = 3.10 (0.03); *F* = 20.10; *p* < 0.0001], and frontal regions [HR = 2.92 (0.01); LR = 3.01 (0.03); *F* = 7.53; *p* = 0.0061]. Due to the highly significant main effect of risk group, most of the interaction effects of risk group were not significant (see Table 3) but most of the comparisons (HR vs. LR) within each of these interaction effects were significant (HR < LR) as shown in Table 4.

**Table 4:** Comparisons between LR and HR groups within the interaction effects of risk group. Significant comparisons (HR < LR) have been marked with asterisks (Sig.: *p < 0.05, **p < 0.01, ***p < 0.001), while the dash sign (-) indicates ‘not significant’. Unrelated factors (cells) have been left blank. [Regions: F = Frontal, C = Central, P = Parietal, O = Occipital; Factors: RG = Risk Group, AG = Age Group, Gen = Gender, Con = Condition].

Effect	Between-subjects factor		Cond (Sig.)		Reg (Sig.)			
	Group (Cond)	Sig.	Loss	Gain	F	C	P	O
**RG × Gen**	Male	***						
**RG × Gen**	Female	*						
**RG × AG**	Younger	***						
**RG × AG**	Older	**						
**RG × Gen × AG**	Younger Male	***						
**RG × Gen × AG**	Older Male	*						
**RG × Gen × AG**	Younger Female	-						
**RG × Gen × AG**	Older Female	*						
**RG × Cond**			***	***				
**RG × Gen × Cond**	Male		***	**				
**RG × Gen × Cond**	Female		*	*				
**RG × AG × Cond**	Younger		***	**				
**RG × AG × Cond**	Older		**	*				
**RG × Gen × AG × Cond**	Younger Male		***	**				
**RG × Gen × AG × Cond**	Older Male		**	-				
**RG × Gen × AG × Cond**	Younger Female		-	-				
**RG × Gen × AG × Cond**	Older Female		-	-				
**RG × Reg**					**	***	***	***
**RG × Gen × Reg**	Male				**	***	***	***
**RG × Gen × Reg**	Female				-	**	**	*
**RG × AG × Reg**	Younger				*	**	***	***
**RG × AG × Reg**	Older				-	***	***	*
**RG × Gen × AG × Reg**	Younger Male				**	**	**	***
**RG × Gen × AG × Reg**	Older Male				-	*	*	*
**RG × Gen × AG × Reg**	Younger Female				-	-	-	-
**RG × Gen × AG × Reg**	Older Female				-	*	*	-
**RG × Cond × Reg**	Loss				**	***	***	***
**RG × Cond × Reg**	Gain				*	***	***	***
**RG × Gen × Cond × Reg**	Male (Loss)				**	***	***	***
**RG × Gen × Cond × Reg**	Male (Gain)				**	**	**	**
**RG × Gen × Cond × Reg**	Female (Loss)				-	*	**	*
**RG × Gen × Cond × Reg**	Female (Gain)				-	**	**	*
**RG × AG × Cond × Reg**	Younger (Loss)				*	**	***	***
**RG × AG × Cond × Reg**	Younger (Gain)				-	**	**	**
**RG × AG × Cond × Reg**	Older (Loss)				-	***	***	*
**RG × AG × Cond × Reg**	Older (Gain)				-	**	**	*
**RG × Gen × AG × Cond × Reg**	Younger Male (Loss)				*	**	**	***
**RG × Gen × AG × Cond × Reg**	Younger Male (Gain)				**	**	*	*
**RG × Gen × AG × Cond × Reg**	Older Male (Loss)				-	**	**	*
**RG × Gen × AG × Cond × Reg**	Older Male (Gain)				-	-	-	-
**RG × Gen × AG × Cond × Reg**	Younger Female (Loss)				-	-	-	*
**RG × Gen × AG × Cond × Reg**	Younger Female (Gain)				-	-	-	-
**RG × Gen × AG × Cond × Reg**	Older Female (Loss)				-	*	*	-
**RG × Gen × AG × Cond × Reg**	Older Female (Gain)				-	*	*	-

Fig 3 illustrates all possible pairwise comparisons (Bonferroni adjusted) between HR and LR groups. It is clearly shown that the HR group has manifested significantly lower theta power than the LR group in both males and females, while the differences were more robust in males, especially in younger males [panel sets A1 and A2]. Specific differences were observed in: (i) younger males during both loss and gain conditions in all scalp regions (*p* < 0.001); (ii) older males during the loss condition at central, parietal, and occipital regions (*p* < 0.001); (iii) younger females during the loss condition only at occipital regions (*p* < 0.05); and (iv) older females during loss and gain conditions at central and parietal regions (*p* < 0.05).

#### Gender

A significant gender × age group interaction (Table 3) suggested that younger males [3.34 (0.03)] had highly significant increases in theta power [*F* = 23.62; *p* < 0.0001] compared to younger females [3.13 (0.03)], while older females [2.88 (0.03)], on the other hand, showed higher theta power [*F* = 4.63; *p* = 0.0315] than older males [2.78 (0.03)]. A significant gender × condition interaction revealed that a significant gender difference [*F* = 8.34; *p* = 0.0039] was observed only in the loss condition, where males [3.03 (0.03)] showed higher theta power than females [2.93 (0.02)]. Gender × condition × region interaction effect revealed that males had higher theta power than females only during the loss condition at frontal [male = 3.05 (0.03); female = 2.92 (0.03); *F* = 10.73; *p* = 0.0011] and central regions [male = 3.05 (0.03); female = 2.92 (0.03); *F* = 11.38; *p* = 0.0008]. Further, this 3-way interaction suggested that males showed higher theta power during loss compared to gain (loss > gain) in frontal regions [loss = 3.05 (0.03); gain = 2.94 (0.03); *F* = 21.04; *p* < 0.0001] and an opposite pattern (gain > loss) in central [loss = 3.05 (0.03); gain = 3.10 (0.03); *F* = 6.00; *p* = 0.0144], parietal [loss = 3.05 (0.03); gain = 3.18 (0.03); *F* = 38.42; *p* < 0.0001], and occipital region [loss = 2.98 (0.03); gain = 3.12 (0.03); *F* = 53.43; *p* < 0.0001]. On the other hand, the same 3-way interaction revealed that females showed significantly more theta power for gain than for loss (gain > loss) in central [loss = 2.92 (0.03); gain = 3.10 (0.03); *F* = 77.87; *p* < 0.0001], parietal [loss = 2.98 (0.03); gain = 3.17 (0.03); *F* = 94.52; *p* < 0.0001], and occipital regions [loss = 2.90 (0.03); gain = 3.10 (0.02); *F* = 108.93; *p* < 0.0001]. Pairwise comparison across gender (male vs. female) shown in Fig 3 [panel sets B1 and B2] revealed more specific findings. Younger males (both HR and LR groups) displayed significant increases in theta power compared to younger females [left column of panel sets B1 and B2]. On the other hand, older females, especially the HR females during the gain condition, exhibited significantly increased theta power compared to their male counterparts [right column of panel set B2].

#### Age Group

Main effect of age group, as shown in Table 3, indicated that younger subjects [3.24 (0.02)] displayed significantly higher theta power [*F* = 163.81; *p* < 0.0001] than older subjects [2.83 (0.02)] in general, and in all or most of the comparisons within the subgroups (see Fig 3, panel sets C1 and C2). Age group × condition effect showed that the younger group displayed significantly higher theta power during loss [younger = 3.21 (0.02); older = 2.76 (0.03); *F* = 173.85; *p* < 0.0001] as well as during the gain condition [younger = 3.26 (0.02); older = 2.90 (0.03); *F* = 117.33; *p* < 0.0001]. The same interaction effect also revealed that significant condition differences (gain > loss) were more robust in the older age group [*F* = 68.21; *p* < 0.0001] than in the younger group [*F* = 13.30; *p* = 0.0003]. On the other hand, age group × region interaction effect showed that while the difference in theta power between the younger and the older age group was significant in all four regions (younger > older), the highest age difference was observed in the occipital region [younger = 3.26 (0.02); older = 2.79 (0.03); *F* = 194.06; *p* < 0.0001], followed by parietal [younger = 3.32 (0.02); older = 2.87 (0.03); *F* = 160.56; *p* < 0.0001], central [younger = 3.22 (0.02); older = 2.86 (0.03); *F* = 103.91; *p* < 0.0001], and frontal region [younger = 3.14 (0.02); older = 2.79 (0.03); *F* = 100.49; *p* < 0.0001]. The pairwise comparisons, shown in Fig 3 [panel sets C1 and C2], revealed that age differences in theta power were highly significant (younger > older) within each gender and risk group during the loss as well as the gain condition, and that these differences were more robust in males compared to females.

#### Within-Subjects Factors

Main effect of condition showed that the gain condition [3.08 (0.02)] showed significantly higher theta power [*F* = 73.31; *p* < 0.0001] than the loss condition [2.98 (0.02)] (Table 3). The region main effect revealed that theta power significantly [*F* = 61.07; *p* < 0.0001] varied across the regions: parietal [3.10 (0.02)] > central [3.04 (0.02)] > occipital [3.02 (0.02)] > frontal region [2.96 (0.02)]. Further, condition × region interaction indicated that the loss condition had higher theta power than the gain condition only in the frontal region [loss = 2.98 (0.02); gain = 2.95 (0.02); *F* = 6.03; *p* = 0.0142], while the reverse (gain > loss) was true for central [loss = 2.98 (0.02); gain = 3.10 (0.02); *F* = 61.11; *p* < 0.0001], parietal [loss = 3.02; gain = 3.17; *F* = 124.72; *p* < 0.0001], and occipital regions [loss = 2.94; gain = 3.11; *F* = 155.45; *p* < 0.0001].

**Fig 4:**
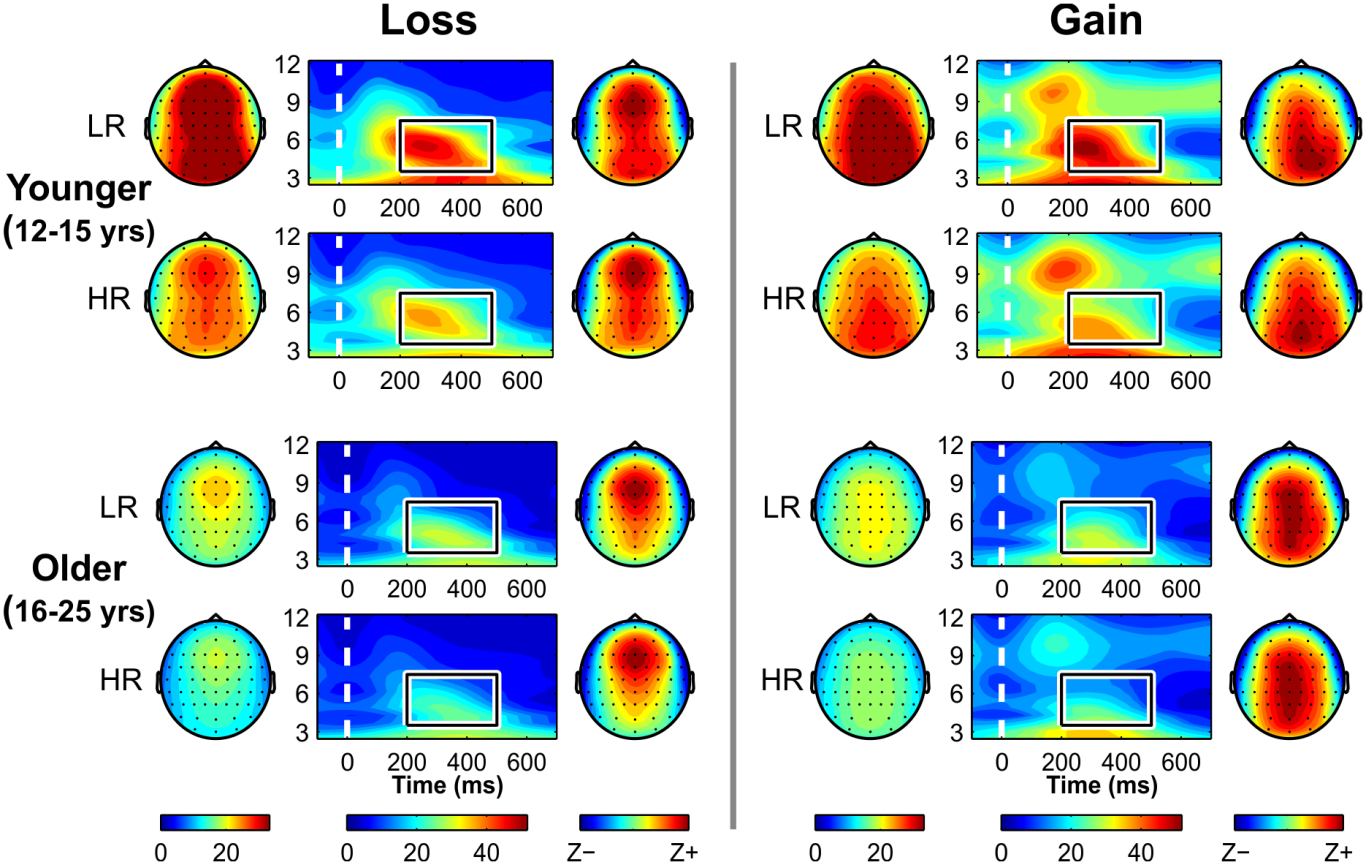
Time-frequency (TF) plots of theta power. TF plots during loss at FZ electrode (middle panels on the left side) and gain conditions at PZ electrode (middle panels on the right side) for the younger (top two rows) and older male subjects (bottom two rows) are shown. Theta power differences between HR and LR subjects (HR < LR) were more robust in males (as shown in these plots) compared to females (not shown). In these panels, each TF plot is flanked by a topographic map of absolute values on the left side and z-scored values on the right side. The rectangle box inside the TF plots represents the Time Frequency Region of Interest (TFROI) of ERO theta band (3.5–7.5 Hz, *y*-axis) within 200–500 ms (*x*-axis). Loss condition with anterior maximum and gain condition with posterior focus are shown. Older groups displays more anterior activity than younger groups (“frontalization”).

Fig 4 shows time-frequency plots and topographic head maps for the younger and older male subjects. Topographically, theta power during loss condition showed an anterior focus while the gain condition had a posterior focus in younger subjects and a wider activation involving frontal-central-parietal regions was seen in older subjects. HR subjects showed significantly lower theta power than LR individuals. Overall, the older subjects have shown lower theta power compared to their younger counterparts in each subgroup.

### CSD Topography

**Fig 5:**
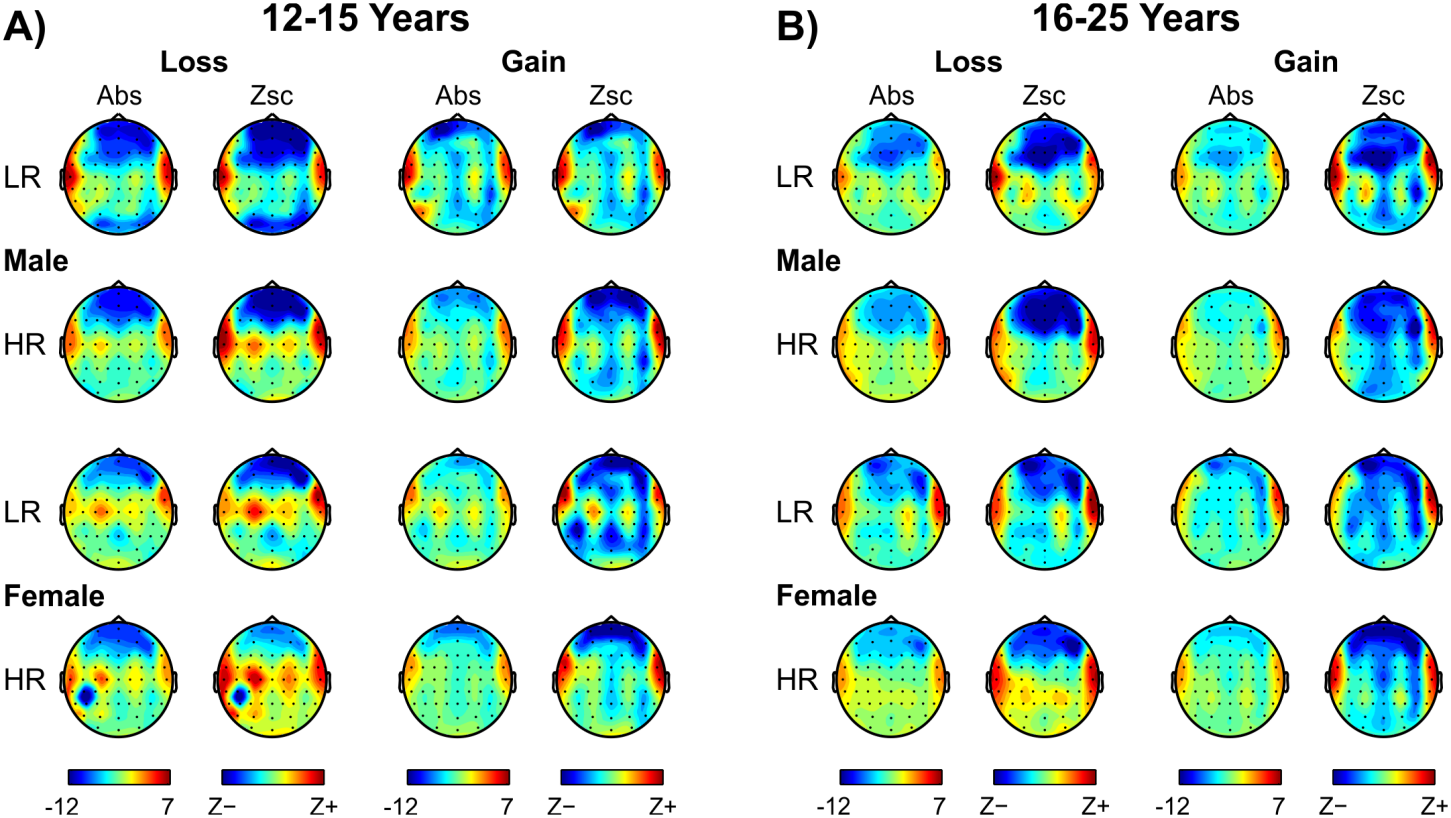
Topographic maps of ERO theta activity. The maps illustrate absolute (Abs) and Z-scored (Zsc) CSD potentials across risk groups for the loss (left columns of panelset A) and gain condition (right columns of panelset B) in the younger (panelset A) and older group (panelset B). Uniform color scales for the absolute maps (μV/cm^2^) and for the Z-scored maps (from minimum to maximum) are shown at the bottom.

CSD topographic maps of ERO theta activity are shown in Fig 5. During the loss condition, bilateral temporal sources in younger HR males were relatively weaker compared to their LR counterparts, while there was no prominent difference between risk groups in younger females. During gain condition, younger HR males showed lower CSD potential in bilateral sources as well as in frontal sinks, while females did not show any marked differences. Among the older groups, both male and female HR subjects displayed weaker frontal sinks than their LR counterparts during loss and gain conditions.

### Externalizing Disorders and BIS Impulsivity

**Fig 6:**
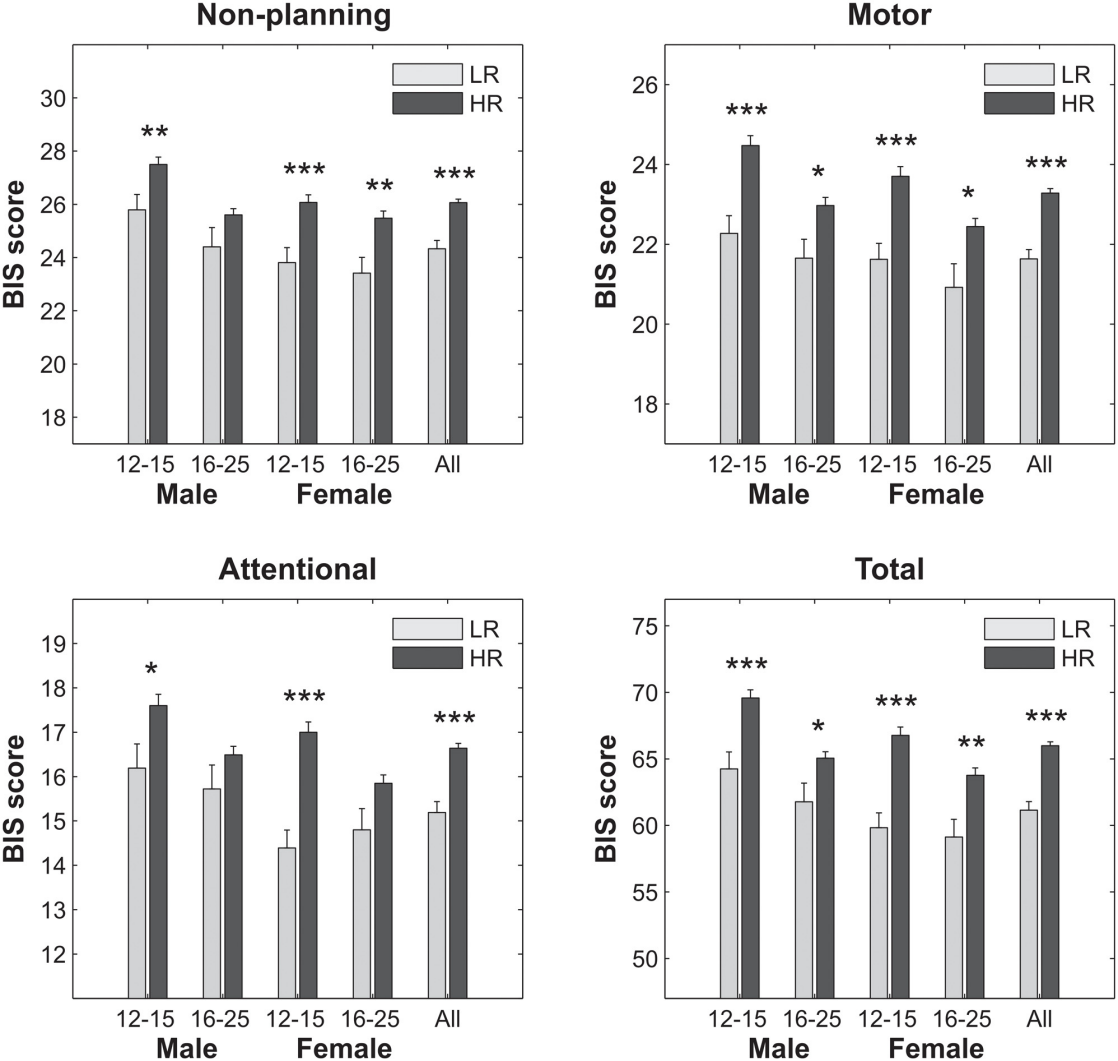
Comparison of impulsivity scores between LR and HR across subgroups. The mean and standard error (SE) of the BIS scores have been plotted. The error bars represent 1 SE. The significance levels have been marked with asterisks (*p < 0.05; **p < 0.01; ***p < 0.001).

As shown in Table 2, prevalence rates were significantly higher in HR compared to LR subjects in several diagnoses (especially for substances such as alcohol, tobacco, marijuana use, as well as for CD and any EXT diagnosis) in males and/or females and in the combined sample. BIS impulsivity scores between LR and HR groups across gender and age groups are shown in Fig 6. Overall, the HR subjects showed significantly increased impulsivity on each of the subscales of BIS (attentional, motor, and non-planning) as well as in total impulsivity in each subgroup. Specifically, younger HR males exhibited significantly higher impulsivity in all subscales and total score than the LR counterparts. On the other hand, older HR males showed higher impulsivity in motor and total score compared to the LR subjects. Further, all BIS scores were significantly higher in younger HR females compared to the LR subjects. Finally, compared to their LR counterparts, older HR females showed significantly increased BIS scores for non-planning, motor and total impulsivity. However, correlations between BIS impulsivity scores and theta power were not significant and had very small ‘r’ values (ranging between 0.005 and 0.056).

## Discussion

The major objective of the current study was to examine neurocognitive deficits during reward processing as measured by ERO theta power in HR offspring from families densely affected by alcoholism compared to LR subjects from comparison (community) families. Theta power, CSD, and behavioral impulsivity were compared between the risk groups in the context of gender and age group. The major findings of the present study are as follows:

1. *ERO theta between risk groups*: HR subjects showed significantly lower theta power than LR subjects (HR < LR), and the differences were more robust among males;
2. *CSD between risk groups*: Differences in CSD topography (HR < LR) were also observed in HR subjects, especially in males, compared to their LR counterparts;
3. *ERO theta between age groups*: Younger subjects manifested significantly higher theta power than their older counterparts consistently across groups and conditions. Further, older subjects displayed relatively more theta activity at anterior sites than younger subjects (“frontalization”) during the loss condition;
4. *ERO theta between genders*: Males displayed increased theta power than females during younger ages (during loss and gain), while females manifested augmented theta power than males (during the gain condition).;
5. *BIS scores between risk groups*: HR subjects as a whole and in several subgroups showed increased impulsivity scores compared to their LR counterparts (17 out of 20 comparisons were significant).

### Lower Theta Power and CSD in HR Subjects

The major finding of the present study is that HR subjects displayed lower theta power compared to LR participants across several comparisons (see Figs 3 and 4 and Table 4). Specifically, as shown in Fig 3, compared to the LR subjects, the HR subjects from younger male and older female subgroups showed lower theta power during loss and gain conditions, while the older HR males and younger HR females showed a decrease only during the loss condition. Further, these differences between the risk groups were more robustly displayed by male compared to female subjects. To our knowledge, this is the first study that has examined reward related ERO theta in HR subjects during a monetary gambling paradigm. This finding of lower ERO theta power during reward processing in HR subjects can be viewed in the light of previous reward related ERP studies which have reported lower amplitudes in N2 during Balloon Analogue Risk Task [24] and in P3 during a visual discrimination task [26]. As the time range of theta power (200–500 ms) used in the present study includes the timing of both N2 and P3 components, and since theta activity is considered to be a major constituent of N2 and P3 [14,69,95], our finding of lower theta power in HR subjects is in line with previous ERP findings during reward processing in similar sample of at risk groups [67]. Although ERO measures, which underlie the P3 wave, have been shown to provide additional information to that offered by conventional ERP amplitude measures [4], comparison of both measures was beyond the scope of the current study.

On the one hand, lower ERO theta power during reward processing observed in this study is suggestive of a dysfunctional reward system in HR subjects. On the other hand, since ERO theta activity has been shown to be related to a variety of behavioral, cognitive, and motivational or emotional aspects of human information processing including reward processing [6,7,11,14,30,39,59,60,109–122], and since HR subjects have been shown to have lower ERO power during other cognitive tasks, such as Go/NoGo [65] and visual oddball [66], our finding of lower theta activation in HR subjects may also indicate a generic cognitive deficit in these ‘at risk’ individuals. However, growing evidence for specific dysfunction in reward processing in HR subjects has been demonstrated by electrophysiological [24,26,67] as well as fMRI studies [123,124]. Since the HR subjects showed lower theta power during both loss and gain conditions, it is reasonable to assume that these ‘at risk’ individuals may have difficulty in evaluating the both positive and negative outcomes. Therefore, this finding may indicate blunted monetary outcome salience or deficient neural reward sensitivity in these subjects. This deficient processing could also be due to general “cortical insufficiency”, a concept initially proposed in developmental dyslexia to explain a deficient processing at specific cortical regions (cf. [125]). In addition, similar to alcoholics, who showed weaker CSD activation during reward processing [49], the findings of the present study demonstrate weaker sources and sinks in HR subjects during reward processing in frontal and temporal regions (Fig 5). As shown by the structural and functional MRI studies, it is possible that brain circuitry underlying reward and emotional processing [126–128] as well as the integrity of white matter pathways are altered in individuals with positive family history for alcoholism [129,130]. Pre-existing cognitive deficits in high risk subjects are also evident from the neuropsychological findings showing compromised executive functioning in these subjects [131–133]. Therefore, it is reasonable to assume that asynchronous (delayed) maturation of prefrontal-limbic circuitry may be involved in the predisposition to develop substance use and related disorders in high risk adolescents and young adults (for a review, see Bava and Tapert [134]).

### Gender Differences in Theta Power

It was found that ERO theta power between HR and LR subjects was statistically significant, and this difference was more robust in males compared to females (Fig 3, Panels A1 and A2). This gender-specific neurophysiological finding in HR subjects is supported by the P3 studies in the literature. For instance, smaller P3 amplitudes in high risk subjects has been reported more often in males [26,135–144] than in females [145,146]. Further, P3 reduction has been shown to be more robust in high risk males than in females (e.g., [147,148]). On the other hand, there are only a few ERO studies on high risk subjects to compare our findings, and gender effect in these studies were either not analyzed [65] or not to be found significant [66]. Nevertheless, it is well-known that the incidence of alcoholism is higher in males than in females, with reports in the United States indicating that both life time and 1-year prevalence rates for alcohol abuse and dependence were more than 2 times higher for males compared to the females [149]. Our finding implicating HR males as biologically more vulnerable compared to the HR females may be partially explained by gender differences in the heritability of alcohol problems [150] as well as in the patterns of transmission of alcoholism between family members [151].

Another gender related finding was that while males produced relatively more theta power (male > female) at younger ages (during both loss and gain), the reverse pattern (female > male) was observed in the older age group (more apparent during the gain condition). (Fig 3, Panels B1 and B2). Similar to this finding, Chorlian et al. [46] reported that both auditory and visual theta EROs were relatively higher in 12–15 year males (than females) while theta power in females more steadily increased (than males) at/after 15 years of age until 25 years. Previous studies from our group have also found that adult females have higher P3 amplitude [152–154] and increased ERO delta and theta power compared to males in cognitive and emotional paradigms [155], including the gambling paradigm [14,41]. More studies on gender differences are needed to confirm our findings as the ERP/ERO studies showing gender differences across development are rare, although gender differences in ERO responses in different frequency bands to cognitive and emotional processing have been widely documented in adult samples [155–159]. As noted by Jausovec and Jausovec [158], gender differences in ERO during visual processing could be explained by evolutionary theories of human visuospatial sex differences [160,161]. This observation could be further reinforced by the evidence for gender differences in anatomical and functional features of the human brain [162–165]. Further studies examining developmental trajectories of reward-related ERO theta power in adolescence and young adults of both genders may shed further light to explain our findings.

### Theta Power between Age Groups

Our results show a significant and robust decrease of ERO theta power with age, presumably reflecting developmental changes in the neural mechanisms of outcome monitoring (Fig 3 Panels C1 and C2). This finding is in line with well-known developmental reduction of P3 amplitude [166,167] and resting EEG power [168–174]. In error monitoring paradigms, the error-related P3 amplitude (“error positivity”) decreased with age [175], in contrast to the error-related negativity (ERN) that showed a significant increase from 7 to 17 years of age [176]. Although there have been no reward related ERO findings reported between different age groups, Hammerer et al. [177] measured the FRN in children, adolescents, younger adults, and older adults, and found that the amplitude of the FRN after gains and losses decreased monotonically from childhood to old age. These developmental changes could be due to dopaminergic and prefrontal contributions to reward-based learning and outcome monitoring across development from childhood to old age [177,178], and may also reflect changes associated with the salience of reward information as well as the declining ability to monitor and control behavior across the life span [179]. In an fMRI task comparing age groups during reward processing, Bjork et al. [180] found that compared with young adults (22–28 years of age), adolescents (12–17 years of age) displayed less recruitment of the right ventral striatum and right-extended amygdala while anticipating responding to gains. Recent fMRI studies have further confirmed differences in reward processing mechanisms between adolescents and adults [181–183].

The current study also indicates topographic changes in ERO theta oscillations during reward processing in adolescent brain development (see Panels C1 and C2 of Figs 3 and 4) comparing adolescent to young adult subjects. Although both older and younger groups manifested anterior maxima of theta power for the loss condition and posterior maxima for the gain condition in line with our earlier study [49], the topography of theta activity in the current study showed increased anteriorization (and decreased posterior activity) during development from adolescence to young adulthood. Gasser et al. [184] studied topographic aspects of EEG development of normal children and adolescents from 6 to 17 years, and reported that the maturation of low frequencies (specifically, theta and alpha bands) started at posterior regions and terminated at anterior derivations with maturation. Further concurring with developmental ERO studies in the literature [52,58,185–188], our finding corroborated the phenomenon of a gradual shift towards anteriorized topography and greater frontal activation in theta activity (i.e., “frontalization”) [189,190], whereby the prefrontal cortex progressively matures and assumes greater control over neural processing from childhood through adolescence to adulthood by virtue of a posterior to anterior progression during brain development [191,192]. According to Gogtay et al. [191], higher-order association cortices (e.g., the prefrontal cortex), mature only after lower-order structures (e.g., somatosensory and visual cortices) in a parietal-to-frontal direction, and phylogenetically older brain areas (e.g., basal ganglia and the limbic system) mature earlier than newer ones (e.g., the prefrontal cortex). Frontalization has also been observed in resting EEG, which corresponds to grey matter development, showing relative power distributed as a function of age with posterior regions maturing earlier than anterior regions (cf. Segalowitz et al. [193]). Specifically, theta-alpha maturation of resting EEG occurs first in occipital regions and then progresses gradually to frontal regions [170,172,194]. These electrophysiological and neuroimaging studies support our finding of more anteriorized theta activation in older subjects compared to the younger group.

### Impulsivity, Externalizing Disorders, Brain Development and Risk for Alcoholism

The HR subjects in general showed increased impulsivity on all subscales and total score of the BIS compared to the LR group, although subgroup-specific findings (i.e., age group and gender related) were also present (Fig 6). Studies have reported higher impulsivity scores and increased prevalence of externalizing disorders in general, and in particular substance use disorders [195–198]. It is also interesting to observe that while younger subjects of both genders showed significant differences between risk groups on all subscales of the BIS, older females displayed significant differences in nonplanning and motor impulsivity, while the older males were significantly different only for motor impulsivity. Similar to our findings, age and gender differences in impulsivity have been reported in the literature [199,200]. It has also been reported that the association between gender and risk for alcohol problems may be mediated by impulsivity [200]. Further, etiological connections between impulsivity and alcoholism and/or other SUDs have also been proposed (for reviews, see [201–203]). Consistent with this view, the current study has also found that the prevalence rate of externalizing disorders was significantly higher in the HR compared to the LR group (Table 2). Importantly, the prevalence rates of externalizing disorders in subjects with family history of alcoholism are higher than normal population levels [76,204,205].

Impulsivity involves actions and tendencies that are poorly conceived, premature, unduly risky and often inappropriate in a given context [206], and by extension is related to hyper-sensitivity to immediate reward (delay discounting), the inability to inhibit pre-potent responses (response disinhibition), and risk taking [207]. Impulsivity includes deficits in attention, lack of reflection and/or insensitivity to consequences, all of which occur in addiction (cf. [208]). Evidence suggests that limitations in brain development during adolescence restrict the ability to control impulsivity and may lead to substance use and addiction [209]. Dysfunctional frontal executive functions have often been linked to impulsivity [208] and substance dependence [210]. According to de Wit [207], impulsivity is both a determinant and consequence of drug use. Further, causal connections linking impulsivity, brain development, and risk for SUDs and/or externalizing disorders have been proposed (for reviews, see [202,208,209]). Taken together, the findings of the present study lend support to these etiological propositions. In other words, each of our findings—decreased theta power reflecting possible cortical insufficiency during reward processing along with increased impulsivity and higher rate of externalizing disorders in HR subjects—may be inherently related to each other in causing and/or maintaining the vulnerability or risk status for alcoholism and other related disorders.

However, it should also be mentioned that correlations between ERO theta power and BIS impulsivity scores were not significant, although such a relationship has been well established in alcoholic subjects [49]. This suggests that neural mechanisms (or circuits) underlying reward processing on the one hand, and that of impulsivity on the other hand, may each be separately related (“parallel connections”) to risk status without influencing each other at relatively younger ages (HR sample) than in older age groups (alcoholic sample). Another possibility is that only those with significant correlations between (and thereby interaction across) these two systems related to reward theta EROs and impulsivity may develop alcohol dependence, although we do not have any evidence in the current study to support this assertion. In a previous study in alcoholics, we found a significant correlation between theta ERO during reward processing and impulsivity (BIS) [49]. This lack of correlation between theta ERO and impulsivity in the current study may be attributable to the differences in brain maturation and reward processing mechanisms between adult alcoholics and the relatively younger HR subjects [181,182]. Geier and Luna [211] report that adolescents relative to adults demonstrated decreased anticipatory processing and assessment of risk, but an increased consummatory response, leading to suboptimal representations of reward valence and decision-making. It is also possible that theta power during the feedback of loss/gain was not inherently related to impulsivity in the younger sample as used in our study, but rather theta power underlying (impulsive) decision making (which the current study has not analyzed) may be more directly related to impulsivity. Further, similar to our results, Bernat et al. [45] did not find a negative relation between ERO theta activity underlying reward processing and externalizing proneness in a sample of university undergraduates, while such a relationship has been reported for the error paradigm (ERN) in a similar sample [212]. Bernat et al. [45] explained that this seemingly inconsistent finding is perhaps due to a functional differences between the components, such that the ERN reflects endogenous representations while the feedback related P3 reflects exogenous cues. While reward theta EROs and risk status may have bidirectional influence with each other, externalizing disorders, which are known to have impulsivity as a core factor, were more prevalent in the HR subjects relative to the LR group, suggesting that both trait impulsivity measured by the BIS and the clinical manifestations of impulsivity (in the form of externalizing disorders) may also be intricately linked to the risk status associated with AUD. Although these explanations might hold true for the results of the current study, future studies may further explore this issue.

### Limitations and Future Directions

The current study has successfully elicited deficient ERO theta power and CSD along with heightened impulsivity in HR subjects; yet caution is advised as there are some limitations: For instance, (i) the sample sizes for the LR groups are smaller than the HR groups; (ii) the trials involving both low and high bet amounts (10¢ and 50¢) have been combined for loss and gain conditions in order to maximize trial numbers and sample sizes; and (iii) other frequencies of EROs, which have not been analyzed (based on our previous studies), may yield additional information. Nevertheless, the findings of the present study may have considerable implications to further characterize neurocognitive dysfunction in alcoholism and risk status.

We suggest that future studies overcome the limitations of the present study to further advance the field, by attempting: (i) to examine the brain circuitries underlying reward/outcome processing in high risk individuals by using coherence/synchrony measures of electrophysiology and by using functional connectivity measures of neuroimaging; (ii) to examine the trajectories of reward-related ERO activity in HR subjects and to delineate longitudinal changes associated with ERO measures in HR subjects using multiple assessments over a period of time; (iii) to extend the study to examine EROs in several externalizing disorders (considered as a spectrum) along with alcohol use disorders; (iv) to include multiple measures of impulsivity and externalizing features in AUD and HR subjects to understand the complex interactions among these factors; (v) to examine, in addition to the outcome processing analyzed in the current study, the decision making aspect, which might prove to be more sensitive in younger HR samples; (vi) to compare the absolute ERO power with the baseline normalized power and to utilize possible complementary merits in both approaches, (vii) to study the effects of genetic variants on trajectories of electrophysiological phenotypes during adolescent and young adult development in high risk and low risk samples; and (viii) to parse out the influence of state (e.g., quantity/frequency of alcohol intake) and trait factors (e.g., impulsivity) in order to measure the distinct as well as the relative contributions of these factors.

## Conclusions

The current study has examined ERO theta power underlying reward processing (i.e., during evaluation of loss/gain outcomes) as well as impulsivity features in HR offspring from high density alcoholism families of COGA and in LR individuals from community families. HR male subjects showed significantly lower ERO theta power and deficient CSD activity during reward processing compared to LR subjects. HR subjects also manifested increased impulsivity and higher rates of externalizing disorders than LR subjects. This lower ERO theta activity during reward processing in HR may reflect neurocognitive deficits that may underlie heightened impulsivity, and increased rates of externalizing disorders, that are inherently related to vulnerability for SUDs. It is suggested that ERO theta power during reward processing may be a useful endophenotype in predicting risk for developing alcoholism and related disorders, and studies are underway to measure the effects of genotypes and phenotypes as well as their interaction in causing specific clinical outcomes.

## Supporting Information

S1 Table Mean (M) and standard deviation (SD) of log-transformed ERO theta power values stratified for risk group, gender, age group, task condition, and scalp region. (DOCX)
