# Supplementary material for: Deficient Event-Related Theta Oscillations in Individuals at Risk for Alcoholism: A Study of Reward Processing and Impulsivity Features
Source: PLoS One. 2015 Nov 18;10(11):e0142659. doi: 10.1371/journal.pone.0142659 (PMC4651365; doi:10.1371/journal.pone.0142659)
Supplement: S1 Table — (DOCX) [file pone.0142659.s001.docx]

S1 Table. Mean (M) and standard deviation (SD) of log-transformed ERO theta power values stratified for risk group, gender, age group, task condition, and scalp region.

| **Risk**  **Group** | **Gender** | **Age**  **Group** | **Loss** | | | | | | | | **Gain** | | | | | | | |
| --- | --- | --- | --- | --- | --- | --- | --- | --- | --- | --- | --- | --- | --- | --- | --- | --- | --- | --- |
|  |  |  | **Frontal** | | **Central** | | **Parietal** | | **Occipital** | | **Frontal** | | **Central** | | **Parietal** | | **Occipital** | |
|  |  |  | **M** | **SD** | **M** | **SD** | **M** | **SD** | **M** | **SD** | **M** | **SD** | **M** | **SD** | **M** | **SD** | **M** | **SD** |
| **LR** | **Male** | **12-15** | 3.41 | 0.63 | 3.42 | 0.57 | 3.49 | 0.62 | 3.46 | 0.58 | 3.30 | 0.67 | 3.46 | 0.59 | 3.56 | 0.57 | 3.50 | 0.58 |
| **LR** | **Male** | **16-25** | 2.84 | 0.55 | 2.89 | 0.55 | 2.85 | 0.55 | 2.75 | 0.58 | 2.73 | 0.62 | 2.91 | 0.56 | 2.97 | 0.60 | 2.89 | 0.53 |
| **LR** | **Male** | **Total** | 3.14 | 0.66 | 3.17 | 0.62 | 3.18 | 0.67 | 3.12 | 0.68 | 3.03 | 0.70 | 3.20 | 0.63 | 3.28 | 0.65 | 3.21 | 0.64 |
| **LR** | **Female** | **12-15** | 3.05 | 0.59 | 3.08 | 0.60 | 3.22 | 0.66 | 3.18 | 0.61 | 3.00 | 0.57 | 3.19 | 0.56 | 3.34 | 0.58 | 3.28 | 0.58 |
| **LR** | **Female** | **16-25** | 2.84 | 0.60 | 2.87 | 0.61 | 2.89 | 0.64 | 2.75 | 0.61 | 2.93 | 0.53 | 3.13 | 0.52 | 3.13 | 0.53 | 3.00 | 0.50 |
| **LR** | **Female** | **Total** | 2.97 | 0.60 | 3.00 | 0.61 | 3.09 | 0.67 | 3.01 | 0.64 | 2.98 | 0.56 | 3.17 | 0.54 | 3.26 | 0.57 | 3.17 | 0.56 |
| **LR** | **Total** | **12-15** | 3.20 | 0.63 | 3.22 | 0.61 | 3.33 | 0.66 | 3.29 | 0.62 | 3.12 | 0.63 | 3.30 | 0.58 | 3.43 | 0.58 | 3.37 | 0.59 |
| **LR** | **Total** | **16-25** | 2.84 | 0.57 | 2.88 | 0.58 | 2.87 | 0.60 | 2.75 | 0.59 | 2.83 | 0.58 | 3.02 | 0.55 | 3.05 | 0.57 | 2.95 | 0.51 |
| **LR** | **Total** | **Total** | 3.04 | 0.63 | 3.08 | 0.62 | 3.13 | 0.67 | 3.06 | 0.66 | 3.00 | 0.63 | 3.18 | 0.59 | 3.27 | 0.61 | 3.19 | 0.60 |
| **HR** | **Male** | **12-15** | 3.22 | 0.60 | 3.19 | 0.60 | 3.24 | 0.63 | 3.14 | 0.60 | 3.09 | 0.62 | 3.25 | 0.58 | 3.37 | 0.60 | 3.32 | 0.56 |
| **HR** | **Male** | **16-25** | 2.71 | 0.60 | 2.67 | 0.59 | 2.63 | 0.58 | 2.56 | 0.56 | 2.65 | 0.60 | 2.78 | 0.58 | 2.82 | 0.55 | 2.76 | 0.52 |
| **HR** | **Male** | **Total** | 2.93 | 0.65 | 2.90 | 0.65 | 2.89 | 0.67 | 2.81 | 0.65 | 2.84 | 0.65 | 2.98 | 0.62 | 3.06 | 0.63 | 3.01 | 0.61 |
| **HR** | **Female** | **12-15** | 3.03 | 0.56 | 3.02 | 0.60 | 3.12 | 0.64 | 3.03 | 0.65 | 3.02 | 0.57 | 3.12 | 0.61 | 3.23 | 0.62 | 3.19 | 0.61 |
| **HR** | **Female** | **16-25** | 2.77 | 0.63 | 2.71 | 0.62 | 2.70 | 0.60 | 2.65 | 0.59 | 2.84 | 0.63 | 2.93 | 0.59 | 2.97 | 0.57 | 2.92 | 0.54 |
| **HR** | **Female** | **Total** | 2.88 | 0.61 | 2.84 | 0.63 | 2.88 | 0.65 | 2.82 | 0.64 | 2.92 | 0.61 | 3.01 | 0.61 | 3.08 | 0.61 | 3.04 | 0.58 |
| **HR** | **Total** | **12-15** | 3.12 | 0.59 | 3.10 | 0.60 | 3.18 | 0.64 | 3.09 | 0.63 | 3.06 | 0.60 | 3.18 | 0.60 | 3.30 | 0.62 | 3.25 | 0.59 |
| **HR** | **Total** | **16-25** | 2.74 | 0.62 | 2.69 | 0.60 | 2.66 | 0.59 | 2.61 | 0.58 | 2.74 | 0.62 | 2.86 | 0.59 | 2.90 | 0.57 | 2.84 | 0.54 |
| **HR** | **Total** | **Total** | 2.91 | 0.63 | 2.87 | 0.64 | 2.89 | 0.66 | 2.82 | 0.64 | 2.88 | 0.63 | 3.00 | 0.62 | 3.07 | 0.62 | 3.02 | 0.60 |
| **Total** | **Male** | **12-15** | 3.26 | 0.61 | 3.24 | 0.60 | 3.29 | 0.63 | 3.20 | 0.61 | 3.13 | 0.64 | 3.28 | 0.58 | 3.40 | 0.60 | 3.36 | 0.57 |
| **Total** | **Male** | **16-25** | 2.73 | 0.59 | 2.70 | 0.59 | 2.66 | 0.58 | 2.59 | 0.57 | 2.66 | 0.60 | 2.79 | 0.58 | 2.84 | 0.56 | 2.78 | 0.53 |
| **Total** | **Male** | **Total** | 2.97 | 0.65 | 2.94 | 0.65 | 2.94 | 0.68 | 2.86 | 0.66 | 2.87 | 0.66 | 3.01 | 0.63 | 3.09 | 0.64 | 3.04 | 0.62 |
| **Total** | **Female** | **12-15** | 3.03 | 0.57 | 3.03 | 0.60 | 3.14 | 0.64 | 3.07 | 0.64 | 3.02 | 0.57 | 3.14 | 0.60 | 3.25 | 0.62 | 3.21 | 0.60 |
| **Total** | **Female** | **16-25** | 2.78 | 0.62 | 2.73 | 0.62 | 2.72 | 0.61 | 2.66 | 0.59 | 2.85 | 0.62 | 2.96 | 0.59 | 2.99 | 0.57 | 2.93 | 0.53 |
| **Total** | **Female** | **Total** | 2.90 | 0.61 | 2.87 | 0.63 | 2.92 | 0.66 | 2.85 | 0.65 | 2.93 | 0.60 | 3.04 | 0.60 | 3.11 | 0.60 | 3.06 | 0.58 |
| **Total** | **Total** | **12-15** | 3.14 | 0.60 | 3.13 | 0.61 | 3.21 | 0.64 | 3.13 | 0.63 | 3.07 | 0.60 | 3.20 | 0.60 | 3.32 | 0.61 | 3.28 | 0.59 |
| **Total** | **Total** | **16-25** | 2.75 | 0.61 | 2.71 | 0.60 | 2.69 | 0.59 | 2.62 | 0.58 | 2.76 | 0.62 | 2.88 | 0.59 | 2.92 | 0.57 | 2.86 | 0.53 |
| **Total** | **Total** | **Total** | 2.93 | 0.63 | 2.90 | 0.64 | 2.93 | 0.67 | 2.86 | 0.65 | 2.90 | 0.63 | 3.03 | 0.61 | 3.10 | 0.62 | 3.05 | 0.60 |
